# Insights into the Brain: Neuroimaging of Brain Development and Maturation

**DOI:** 10.17756/jnpn.2016-003

**Published:** 2016-03-10

**Authors:** Amanda E. Lyall, Peter Savadjiev, Martha E. Shenton, Marek Kubicki

**Affiliations:** 1 Psychiatry Neuroimaging Laboratory, Department of Psychiatry, Brigham and Women’s Hospital, Harvard Medical School, Boston, MA, USA; 2 Department of Psychiatry, Massachusetts General Hospital, Harvard Medical School, Boston, MA, USA; 3 Department of Radiology, Brigham and Women’s Hospital, Harvard Medical School, Boston, MA, USA; 4 VA Boston Healthcare System, Brockton, MA, USA

**Keywords:** Human brain, Neuroimaging methodologies, Brain development, Gray and white matter, MRI, DTI

## Abstract

The study of how the human brain develops has always been a challenge and an interest to the scientific community. In recent years, new evidence has suggested that many neuropsychiatric disorders may originate from aberrations early in development. This discovery necessitates the application of methodologies that make possible the investigation of human brain development *in vivo* and across the lifespan. In this commentary, we present evidence that the advent of structural neuroimaging has specifically and significantly contributed critical information about the developmental trajectories of postnatal human brain development that would otherwise not have been possible. We believe that this is particularly relevant to present day research as it has become increasingly clear that growth trajectories within the brain might serve as an endophenotype for a number of factors, ranging from IQ to psychiatric illness. We highlight seminal early works that helped to jumpstart the field of developmental neuroimaging and which inspired incredible new advances in neuroimaging methodologies that are being developed and applied in the field today.

## Introduction

Human brain development is a complex and intricate process that starts in the fourth week of gestation and extends throughout the lifespan [1, 2]. The multiple dynamic events that interact and contribute to brain development include molecular, genetic, and environmental factors, which, when combined, lead to the emergence and differentiation of neuronal structures that are later shaped to support a fully functional human brain. While the most dramatic brain growth and differentiation occurs prenatally (including the production of neurons, their migration, formation of cortical and subcortical structures and major white matter fiber pathways), it is the early postnatal years that are known to be the most critical for brain development [1, 2]. More specifically, the first years of postnatal life bring rapid myelination, pruning, and further brain growth, with the latter reaching 90% of adult volume by the end of the preschool period [3, 4]. Brain development following these early years is dedicated to improving and to optimizing neuronal function, and, eventually, to protection against cellular damage and tissue deterioration during aging [1, 2].

Precise mapping of longitudinal brain development, including the timing and biological underpinnings of changes along maturational trajectories, is particularly important as there is a general consensus that many psychiatric disorders, for example autism and schizophrenia, may originate from aberrations that take place in the initial stages of brain development [5, 6]. Recent studies, in fact, point to developmental delay, lag, or accelerated deterioration as neurodevelopmental models for various neuropsychiatric disorders [7]. Consequently, attention is shifting towards earlier stages of brain development, before individuals present with clinical symptoms, in the hopes that early intervention might mitigate, or even prevent, the onset of psychiatric illness. However, without an accurate understanding of healthy maturational trajectories, it is difficult to draw accurate conclusions from what one might define as “diverging from normal”.

Prior to the emergence of neuroimaging, post-mortem studies were the only approach to study human brain development. As is apparent, the use of post-mortem brain tissue is not ideal for modeling longitudinal trajectories of age-related brain changes. The small, heterogeneous sample sizes, along with technological challenges related to tissue processing, produced results that were limited by small statistical power. Thus, although these studies were vital to the advancement of neuroscience by yielding groundbreaking information about the complexity and composition of cortical tissue, the adoption of neuroimaging methods proved to be a significant step forward in constructing a clearer picture of the lifetime trajectories of brain development.

The early introduction of such neuroimaging methodologies as Computed Tomography (CT) and transcranial ultrasound reflected a major breakthrough in neurodevelopmental research because they allowed, for the first time, insight into *in vivo* brain structure. These technologies were nonetheless limited in sensitivity and specificity, and in the availability of only gross volumetric measurements. Of note, however, it was the advent of MRI, which followed these earlier methodologies, that led to a rekindled interest in understanding the normative trajectories of human brain growth, and in how this new knowledge might be helpful in identifying abnormalities that appear in individuals with major psychiatric disorders.

The first MRI studies utilized low-field MRI (e.g., 0.5T–1.5T scanners), which produced low-resolution, noisy brain images compared to the images acquired on present day scanners [8]. Nonetheless, these initial MRI scans could differentiate between gray and white matter, as well as separate (also known as parcellation) individual neuroanatomical structures such as the gyri and sulci. These early methods made it possible to make inferences about local brain growth.

Further improvements in MR technology, most notably higher magnetic fields (e.g., modern 3T and 7T scanners), produced images of sub-millimeter resolution, with a high signal-to-noise ratio, thereby enabling a more precise characterization of macrostructural features for even the smallest of anatomical regions. The introduction of additional MR methods has further made it possible to study microstructure in both gray and white matter. These modern neuroimaging methodologies, most particularly higher field MRI and Diffusion Tensor Imaging (DTI) are now powerful tools that accurately describe complex developmental trajectories of the many stages of structural brain development. Thus as is evident from this historical perspective, the application and development of increasingly refined image analysis methods has furthered our overall understanding of how the brain grows.

Another advantage of neuroimaging is its ability to extract and analyze multiple features across the entire brain simultaneously. In addition, because MR is noninvasive, large-population longitudinal studies are possible, which leads to a significant increase in statistical power for neurodevelopmental research. Moreover, the analysis of multiple features such as cortical volume, cortical thickness, and the microstructure of a given white matter tract, are all now possible using advanced image analysis techniques (see below).

While these features can serve as indirect representations of biological mechanisms and events that guide brain development and maturation, the neurobiological specificity of MRI remains limited. The MR signal is averaged over a few cubic millimeters (the resolution of single “voxel”), and is derived from the differential properties of imaged tissue. The amount of “free” and bound water, as well as the macromolecules within the voxel, can influence this signal, making it possible for MR to distinguish tissue containing cellular bodies and processes (gray matter), from tissue that contains myelinated axons (white matter). Further biological differentiation, however, is difficult when scanning *in vivo* subjects. Since, brain tissue is comprised of cell bodies (neuronal and non-neuronal), axons, dendrites, synapses, myelin, vasculature, and extracellular space, it is apparent that these cellular components are too small to study *in vivo* even with the highest spatial resolutions available today on human MRI scanners. *Ex vivo* imaging studies in both human and animal models have demonstrated the possibility of attaining greater image resolutions, which makes possible the investigation of more refined biological elements (e.g., [9]). However, similar to post mortem studies, due to the cross-sectional nature of the *ex vivo* image acquisitions, these studies provide limited information relevant to the underlying processes driving longitudinal maturational trajectories. It is nevertheless believed that the major sources of MR signal that change during brain development include, on the one hand, axonal and synaptic growth (until puberty), followed by synaptic pruning and cell loss, and, on the other hand, myelination growth (which continues until adulthood), followed, by myelin degeneration (with aging). Despite the limitations of *in vivo* neuroimaging, MRI- and DTI- derived parameters, such as cortical thickness or fractional anisotropy (both of which will be discussed in detail later) can serve as indirect indicators of these underlying biological components as they change over the course of the lifespan.

In the following commentary, our intention is to present evidence supporting the opinion that the evolution of MR structural neuroimaging measures has helped the scientific community to achieve a greater understanding of postnatal anatomical human brain development that would not otherwise have been possible. We decided to take a historical approach to demonstrate the influence that neuroimaging has made on the field by first introducing earlier methods and then proceeding to showcase some of the extraordinary new advances in neuroimaging methodologies that are being developed and applied in the field today. This commentary is separated into two primary sections: gray matter and white matter. As there are a significant number of studies, especially in the postnatal imaging field, which have studied human brain development with MR structural neuroimaging measures, we chose to only highlight select publications that we felt best represented singular and exemplar examples of the type of image analysis method discussed in the context of human brain development.

## Gray Matter

### Total brain volume

The early application of MR methodologies primarily focused on volumetric analyses of whole brain cortical gray matter. Seminal developmental studies employing volumetric whole brain methods showed that overall total brain volume follows a curvilinear, inverted U-shaped pattern of growth from birth to adolescence, whereby early increases are followed by a gradual decrease with increasing age [10, 11]. However, many of these early studies did not include scans of children younger than 4 years of age. Recent studies by Knickmeyer et al. [3] show that the initial increase in total brain volume after birth is particularly dramatic [3]. It increases roughly 101% within the first year alone. When compared to the adult brain, a 2-week-old human cortex is 36% of adult volume, 72% of adult volume by 1 year, and 83% of adult volume by two years of age [3]. Of further note, peak total brain volume is reported to occur between 12 and 15 years of age, after which it is reported to gradually decrease [4]. Whole brain volumetric studies also demonstrate clear differences between genders, with male total brain volume being roughly 10% larger than female total brain volume [12]. Taken together, these studies demonstrate, *in vivo*, the dynamic growth of overall brain volume in healthy subjects across the lifespan, and they confirm the critical importance of early postnatal years for long-term neurodevelopment.

### Cortical and subcortical gray matter volume

Following the introduction of higher field MR magnets, which provide better contrast and higher spatial resolution, studies began to subdivide the cortex into lobes: occipital, parietal, temporal and frontal. The ability to acquire several hundred MRI scans from healthy individuals who span a wide age range also made it possible to evaluate regional developmental trajectories in brain maturation [10, 11]. Findings from these studies report distinct regional differences in gray matter maturational patterns, confirming previous findings of cross-sectional post-mortem analyses by Huttenlocher and others [13–17]. Additionally, seminal imaging studies by Giedd and colleagues demonstrate that the subdivided cortex still exhibits an inverted-U pattern in both cortical and subcortical gray matter, although this pattern exhibits a posterior-to anterior and a medial-to-lateral pattern of regional growth [10]. This pattern also mirrors the general acquisition of functional processes related to specific cortical regions, such as visual or auditory capabilities [18, 19]. Subcortical areas have also been shown to reach peak volume before cortical areas, and cortical sensory regions reach peak volume earlier than cortical association and higher-cognition areas [10, 11, 20]. Interestingly, in studies focusing on the earliest stages of brain development, Gilmore and colleagues [21] report that primary sensory and motor regions tend to grow less than other regions of the cortex, confirming the notion that these regions actually experience a greater degree of growth prenatally, and reach maturity within the first year of life [3, 14, 21]. Finally, although many of these findings relevant to gray matter maturation are not new, the application of neuroimaging methods to a longitudinal sample of healthy individuals allows us to observe and to quantify developmental trajectories that were heretofore only hypothesized.

### Gyrification

While volumes of gray and white matter undergo dynamic change as a function of brain development, maturation and aging, the cortical folding pattern, or gyrification, is established during the 2^nd^ and 3^rd^ trimester of prenatal growth [22, 23]. This pattern is largely set at birth and does not undergo a large degree of change as people age. Nonetheless, as brain growth progresses, further deepening of the sulci and enlargement of the gyri occur, yet, the pattern of cortical folds does not change. This was recognized as being a useful developmental feature for neuroimaging studies, even at later periods [24–26]. Thus utilizing this measure, any deviations from a normal gyrification pattern would strongly suggest aberrations in fetal brain development, which could be dated back to a particular period of gestation. In fact, clinical studies of infants born with lissencephaly, a genetic disorder where the cortex does not develop the canonical cortical folds, reveals that gyrification patterns may be a useful measure that indicates the presence of aberrant early brain development [27].

This close tie with early development is also why some of the earliest neuroimaging studies chose gyrification patterns as a central focus (e.g., [28–30]). Earlier techniques for the quantification and analysis of gyrification used manually traced contours of the cortical surface in histological sections of post-mortem brains. For example, Zilles et al. defined a gyrification index as the ratio of lengths of the complete and outer (superficially exposed) contours of a histological brain slice [28]. More recently, the introduction of advanced neuroimaging methods have made possible the development of a variety of image-based mathematical tools for cortical surface shape analysis. For example, Yu et al. [31] used spherical wavelets to characterize cortical folding patterns while Germanaud et al. performed a spectral analysis of the spatial frequencies of folding patterns [31, 32]. In another study by Awate et al. [33], the authors utilized descriptors computed from the differential geometry of surface patches to characterize sex differences in gyrification patterns [33]. These measures have been used to test neurodevelopmental hypotheses in neuropsychiatric diseases because it has been suggested that such features might be under tight genetic control and therefore less susceptible to environmental changes. Thus the use of gyrification measures from neuroimaging methods might help in the quest for structural biomarkers related to genetic risk for major psychiatric disorders. In fact, this method has already shown to be effective in identifying differences in the gyral patterns of patients with chronic schizophrenia, specifically in the frontal and temporal lobes [29, 34].

### Cortical thickness and surface area

In parallel with mapping and characterizing cortical gyrification patterns, new measures have been developed to further characterize cortical volumes. The utilization of new measures is important because cortical volume itself does not reflect the true complexity of cortical growth patterns. For this reason image analysis tools were developed to measure the two primary features of cortical volume: cortical thickness and surface area. These tools utilize surface-based representations of the cortical mantle and offer a significantly more biological representation of the cortex. The development and application of surface-based methods now make it possible for previously identified maturational trajectories of cortical volume to be deconstructed into these two components, providing greater biological specificity, as described below.

In biological terms, cortical thickness represents the “height” of the cortical column whereas surface area is the area of the cortical region of interest. From previous histological studies dating back to the early 1900s, cellular composition within the 6-neocortical layers is shown to vary greatly across the cortex (i.e., Brodmann, Von Economo). Mechanical theories of cortical development suggest that radial growth (i.e., cortical thickness) occurs earliest in development [35]. Tangential growth (i.e., surface area), on the other hand, is believed to be the result of a massive proliferation of cortical cellular processes, such as dendrites, axons, synapses, (as well as continued myelination discussed later), and is believed to take place at later stages of postnatal development [35]. Of further note, even without the resolution to assess microstructural changes in the different 6-neocortical layers, cortical thickness and surface area prove to be extremely valuable in understanding maturational trajectories and they confirm early theories about the ways in which the cortex grows [13, 14, 35].

More specifically, the early application of surface-based methods show that cortical thickness and surface area are not uniform across the cortex, thereby confirming previous seminal cytoarchitectural studies, by Brodmann and Von Economo [36, 37]. These imaging studies demonstrate that rates of growth also vary, with isocortical areas (6-layered, most of the neocortex) showing more cubic patterns of development than allocortical areas (3-layered, i.e. cerebellum), which exhibit more linear patterns of development [20]. Similar to cortical volume, cortical thickness and surface area exhibit inverted-U cubic trajectories [38], with cortical thickness reaching peak values around 8 years of age, with no apparent influences by gender [38], and with surface area peaking later in childhood with sexual dimorphism, (8 years of females and 9.3 years in males) [38]. Furthermore, both cortical thickness and surface area exhibit extremely dynamic and regionally heterogeneous patterns of growth within the first two years of life [39]. That is, between birth and 2 years of age, overall cortical thickness increases by an average of 36.1% per region of interest, while surface area increases 114.6% per region of interest. More importantly, by age of 2, cortical thickness is shown to reach 97% of adult values, while surface area only reaches 67% of adult values [39]. These dynamic growth rates in the first two years are followed by much slower growth in the following years, with surface area, rather than thickness, being the principal driving factor in overall cortical volume growth. In fact, recent studies show surface area to be a pivotal feature in individual variation in brain size, IQ prediction, and a key mediator of gray matter deficits in psychiatric disorders, specifically schizophrenia [40–42].

The application of surface-based measures also led to the discovery that cortical thickness and surface area are mediated by distinct, largely non-overlapping genetic components [43, 44]. The genetic independence of these measures allows for greater biological specificity in the identification of factors mediating individual differences in regional cortical thickness or surface area in healthy populations. These imaging measures can also assist in narrowing the field of investigation to genes that may mediate specific pathologies (e.g., specific reductions in cortical thickness but not surface area) within clinical populations.

### Future directions - gray matter

Taken together, it is clear that the application of neuroimaging methods has led to the characterization of *in vivo* longitudinal gray matter developmental and maturational trajectories that would not otherwise have been possible. The dramatic evolution of imaging techniques developed and applied to the study of gray matter growth has led to an increasingly more refined picture of the structural changes that occur after birth and throughout the lifespan. As is evident from the progress made since the first MRI studies, innovative new tools will ultimately bring us closer to interrogating more neurobiological or cellular features with neuroimaging. One example is new cutting-edge technology that addresses one of the most difficult problems in the neuroimaging field: identifying and differentiating neocortical laminae. More specifically, in a recent publication by Barazny and Assaf [45], *in vivo* whole-brain visualization of the 6 cortical layers was accomplished utilizing inversion recovery MRI and the T1-properties of the cortical tissue [45]. Impressively, these findings have a high correspondence with histology within both rat and human cortices [45]. This methodology could provide a powerful tool to study not only the macroscopic organization of the cortex, but also the *in vivo* longitudinal development of the cortical layers in individual subjects. Another example of a novel method introduced to study gray matter development is a diffusion-based measure called Heterogeneity, developed by Rathi et al. [46]. Heterogeneity measures the variability of water diffusion within a specific region of interest, which indirectly reflects the organization of cortical gray matter complexity [46]. This method has already been shown to be useful in identifying retrogenesis of gray matter in a healthy aging population [46]. Although this method has not yet been applied to the study of gray matter development, it offers the ability to study longitudinal *in vivo* changes in cortical complexity and organization, which undergo microstructural changes from early development periods to later life, as shown by previous post-mortem studies [15–17].

## White Matter

The study of white matter is a critical component to understanding lifespan neurodevelopment and brain maturation. Unlike gray matter, which experiences a dramatic increase within the first few years of life, white matter growth exhibits a more gradual pattern of development [3, 47]. In fact, myelination occurs almost entirely postnatally and continues well into the third decade of life [48].

The MR techniques described in the previous section make the visualization and differentiation of both gray and white matter possible. This is achieved via the manipulation of the physical parameters of MR pulses, making MR sequences differentially sensitive to cellular type, density, tissue structure, lipid and water content. These high spatial resolution MR images, coupled with high contrast between gray and white matter, allow for more precise and automated segmentation of the brain. This, in turn, allows for the investigation of both gray matter changes over time, as described in the previous section, and white matter changes over time.

### White matter volume

Early imaging studies showed changes in white matter volume that exhibited different maturational trajectories than those of gray matter. Moreover, these changes in white matter volume reflect specific changes in white matter tissue structure and content. Specifically, white matter contains axons and glial cells, specifically the myelin-producing oligodendrocytes. The former interconnect proximal and distal cortical areas, providing communication within and between large functional networks, which travel in large fascicles or bundles, while the latter provide protection, insulation, and support for axons. Of note, although the growth of neurons is largely finished before birth, glial cells are actively shaping white matter structure and function throughout life, first producing and growing the myelin sheath around the axons, which improves conductivity between cells, and then pruning and removing unnecessary cells and processes, which leads to optimizing brain connections, and finally providing protection from various stressors across the lifespan.

Previous post-mortem studies have shown that central nervous system myelination follows predictable topographical and chronological sequences, with myelination occurring in the proximal pathways before distal pathways, in sensory pathways before motor pathways, in projection pathways before association pathways, in central sites before poles, and in occipital poles before frontotemporal poles [49–51]. The general pattern of adult myelination is present by the end of the second year [52]. MRI studies demonstrate that this pattern is reflected in tissue relaxation times, which increase as myelination progresses. This is due to changes in tissue water content, and increased volume of lipid-containing myelin, which leads to not only improved contrast between gray and white matter, but also to increased volume of white matter itself. The contrast between gray matter and white matter is reversed until 6 months of age, when compared to the adult brain [53]. This is because gray matter is more developed and contains a greater cellular density, which results in a brighter appearance, while white matter, which contains water and non-myelinated axons, appears darker on T1W, structural scans. This contrast diminishes in the first year of life, and reverses to “adult-like” contrast, with darker gray matter and brighter white matter, by the end of the second year of life, which coincides with the myelination of axonal fibers [53].

While white matter reaches “adult-like” MR signal contrast at the age of one, white matter volume does not stop expanding until adulthood. Volumetric imaging studies have demonstrated that this expansion, especially between the ages of 4 and 22, is relatively linear, increasing about 12% per year [10], with boys exhibiting a steeper increase when compared to girls, which is most likely related to testosterone levels [54–56]. Volumetric neuroimaging studies of early postnatal brain development also show that within the first two years of postnatal development, white matter volume increases only 11% in the first year and 19% in the second year, consistent with annual linear increases reported at later ages [3]. However, although early volumetric studies shed light on developmental changes in white matter, it is only following the introduction of Diffusion Tensor Imaging (DTI) that white matter has been placed at the center stage of brain development research.

### Microstructure of white matter

#### Diffusion MRI

Diffusion MRI was first clinically applied in 1986 by Le Bihan and colleagues. It is an advanced imaging method that utilizes the inherent diffusion properties of water molecules in biological tissues to quantify the directionality and amount of diffusion (either restricted or non-restricted) in a given location. Within brain tissue, this is particularly relevant to white matter where the mobility of water is restricted by both the lipid-rich myelin sheath surrounding the axons and by the high density of axons contained within the fiber bundles. In DTI (introduced by [57]), the diffusion information is acquired along several non-collinear directions, and modeled using an ellipsoid, also known as a tensor, that quantifies and characterizes both the orientation and the amount of diffusion within each voxel, and from which a number of measurements can be calculated. The common metrics of white matter microstructure include mean diffusivity (MD)- which reflects overall water diffusion in the voxel, fractional anisotropy (FA)- the most inclusive diffusion measure, reflecting organization, coherence and integrity of white matter within the voxel, axial diffusivity (AD)- measuring diffusion along the principal direction of axon, and believed to be related to axonal integrity, and radial diffusivity (RD)- measuring diffusion perpendicular to the axon, possibly reflecting myelin. While some of these measures have been partially validated using animal models [58–60], there is still some controversy regarding their specificity [60–62]. Here we focus on reports of FA, as this is the most widely used DTI measure in neurodevelopmental studies.

There are a number of DTI studies that focus on lifespan white matter development in humans, most of which have utilized one of the most common tools in DTI, fiber tractography. Tractography utilizes directional information from the diffusion tensor, resulting in the delineation of individual anatomical white matter tracts in individual subjects. Lebel and colleagues [48, 63, 64], for example, have conducted a large number of maturational tractography studies. In these studies, the authors utilize a normative cohort of over 400 individuals, both males and females, with an age range of 5 to 83 years. Using this cohort, they have plotted the maturational trajectories of 12 major white matter tracts. Findings show that the primary diffusion metric, fractional anisotropy (FA), exhibits a tract-specific maturational profile that is in line with previous post-mortem studies [48–50, 65]. These profiles follow Poisson-shaped curves, which are characterized initially by a period of marked incline towards the peak, followed by a more gradual decline. Different white matter tracts are also shown to reach peak FA at varying ages, which is believed to reflect the age at which that tract is mature. The earliest maturing tract is the fornix, which reaches peak FA at or before 20 years of age, while the last tract to mature is the cingulum, which reaches peak FA at or after 40 years of life [48]. These studies also show that there is considerable variation in the peak FA values that are attained, also suggesting regional variability in the size, degree of myelination, or packing density of white matter tracts.

Lebel and colleagues did not, however, show a significant difference in the maturational profiles between males and females, although recent reports show that healthy females tend to have generally lower FA compared to healthy males [66]. Gender differences are also evident in the overall connectivity of the brain, where male brains appear to be more optimized for intra-hemispheric connections, while female brains appear to be optimized for inter-hemispheric connections [67]. As stated previously, the relationship between myelination and testosterone levels are proposed to play a role in this difference, as shown in a study by Herting and colleagues, where testosterone levels predicted higher FA in boys when controlling for age and puberty [68].

Similar to studies of gray matter, many of the larger developmental cohorts did not include the early postnatal years. DTI studies of healthy term neonates report increases in FA values in all regions (except the splenium), with concomitant decreases in mean diffusivity (MD), which are correlated with decreases in the T2 signal but not the T1 signal [69]. In a study by Geng et al. [70], the authors analyzed the developmental trajectories of 10 white matter tracts from birth (2 weeks) to 2 years of age and show that all tracts studied exhibit increases in FA, with greater rates of FA increase in the first postnatal year than the second year [70]. The increased use of diffusion imaging in the early postnatal years also provides important information in the evaluation of premature infants. For example, Hüppi and colleagues show that brain areas with reductions in FA in pre-term infants are associated with perinatal white matter injury, thought to reflect incoherent fiber tract organization within central white matter regions [71].

These studies provide important information regarding the considerable time and differential pattern of white matter maturation throughout the lifespan. Due to the fact that almost all white matter myelination occurs after birth, it has been suggested that white matter may be more susceptible to environmental factors or other insults [72, 73]. For example, Kochonov and colleagues suggest that the developmental onset of many psychiatric disorders, but more specifically schizophrenia, coincides more closely with the maturational peak of white matter [74]. In fact, it has been suggested that myelination may be responsible for the closing of sensitive periods within the brain, whereby cortical connections may become more “hard-wired” and less plastic after they are fully-myelinated [75].

The use of DTI to study white matter neurodevelopmental trajectories thus makes it possible to confirm previous postmortem studies and it also provides a powerful approach to interrogate potential differences in lifespan white matter maturation *in vivo* in both healthy and clinical populations.

### Additional methods to investigate white matter maturation

As discussed previously, neurobiological changes associated with white matter maturation are, for the most part, associated with either axons or myelin. Axonal size, thickness within a bundle, and axonal coherence are all reported to change with development, in addition to myelin thickness and composition. Unfortunately, all of these factors can influence diffusion, and thus FA. More specifically, even though FA has been widely associated in the literature with myelin integrity, only a small percentage of FA changes are explained by myelin [76]. Further, and more specifically, animal models of demyelination show that total loss of myelin accounts for only 16% decrease in FA [76]. There are also reports of FA changes in childhood and adolescence following different trajectories in boys and girls [66], which are not explained solely by myelination. For this reason, while DTI remains the most popular method to investigate white matter microstructure, other methods are used to complement FA. For example, since T2 transverse relaxation times are highly related to tissue composition, and, as discussed previously, T2-weighted gray-white matter contrast dramatically changes within the first year of age, the sampling of T2 decay through T2 relaxometry might provide additional information about the time-course of brain changes as a function of maturation. The T2 relaxometry derived measure- R(2) has been used previously as a proxy of myelination, and appears to be related to motor speed decrease with aging [77]. T2 relaxometry can also be used to distinguish between fast and slow relaxing water pools, with fast relaxing water more strongly associated with myelin component (more specifically water trapped between the myelin sheath layers- also known as myelin water fraction- MWF). The latter finding has been used to demonstrate the trajectory of brain myelination in infancy and early adulthood, with myelination beginning in the cerebellum and internal capsule prior to 3 months of age, then proceeding to the splenium, body, and genu of the corpus callosum, the optic radiations, the occipital and parietal lobes, and finally culminating with the frontal and temporal lobes as the last to myelinate [78].

Besides T2 relaxation, magnetization transfer is another source of information that can be used to increase microstructural specificity of imaging. With the use of additional inversion recovery (IR) pulse that suppresses the signal of water bounded to macromolecules, one can indirectly measure the concentration of these macromolecules in white matter tissue. Magnetization Transfer Ratio (MTR) is positively correlated with myelin content [66], and also decreases with aging [79]. During adolescence, however, MTR decreases, despite the finding of increases in white matter volume in boys [55], suggesting that its trajectory might be different than that of R(2) or FA.

Another measure related to myelin macromolecules, and recently used in the study of brain maturational trajectories across the lifespan (from 7 to 85 years of age-[80]), is a measure of longitudinal relaxation, or quantitative T1 (R(1)). R(1) follows a inverse-U trajectory, with rates of increase during maturation mirroring the rate of decline with aging. T(1) seems to peak later in life than FA (which shows asymmetric trajectory, with rapid growth and slower decline), suggesting that it might reflect different aspects of white matter maturation. While differences in biological specificity of R(1), R(2) and MTR have not been explored, future maturational models of white matter will most likely combine multi-modal imaging information.

### Future directions - white matter

As the field of imaging progresses, in addition to novel acquisition paradigms, new image processing methodologies are being developed to improve signal modeling. It is especially noticeable in the field of diffusion MRI. New acquisition paradigms, such as Diffusion Spectrum Imaging (DSI) [81] or qBall [82], and new reconstruction methods are all leading to significant improvements in white matter structure delineation, resulting in increased biological specificity. One of the more recent improvements is the creation of multi-tensor tractography [83], which is particularly useful in the area of crossing fibers, allowing for more precise anatomical representation of cortical structural connectivity. Another way in which diffusion imaging is pushing the envelope is through the use of multi-compartmental models of the diffusion signal. Methods such as Free Water Imaging [84] and the neurite orientation dispersion and density imaging (NODDI) model [85], both of which treat the diffusion signal as the sum of multiple compartments. For example, in Free Water imaging, the diffusion signal is modeled as the sum of an isotropic compartment, called Free Water, which represents the unbound freely diffusing water in the extracellular space. The residual diffusion signal in this model, called FA-t, is then modeled in a manner similar to a traditional tensor model, but where the improvement lies in the fact that it is now solely drawing signal from the intracellularly bound water both within and surrounding the white matter fiber bundles [84]. NODDI utilizes a similar paradigm, whereby the “cellular” diffusion signal is modeled as two non-linear components: neurite orientation dispersion index (ODI) and neurite density index (NDI). Each of these components is purported to reflect specific microstructural features of the white matter bundles, where ODI reflects axonal organization, and NDI reflects cellular integrity [85]. In a recent publication that utilized NODDI in 66 healthy subjects, ages 7 to 63 years of age, Chang and colleagues [86] report that NDI exhibits striking logarithmic increases with age whereas ODI increases following an exponential pattern. The authors were also able to show that the use of these advanced analytical methods allows for a more precise prediction of chronological age than previous DTI metrics [86]. Finally, while ODI reflects intravoxel organization (or architecture) of axons, other methods have also been developed to model more macroscopic behavior, or architecture, of white matter fiber bundles. As one example, the macrostructural white matter geometry measures, introduced by [87, 88], are designed to track architectonic changes of white matter during brain development, and are useful in detecting developmental abnormalities in diseases such as schizophrenia and autism [88, 89].

## Conclusions

In summary, the evolution and application of neuroimaging methodologies have made significant contributions to our understanding of lifespan trajectories of human brain development. This remarkable increase in knowledge cannot be understated. As evidenced by the studies highlighted above, these technological advances in imaging have led to discoveries that now allow us to quantify, to characterize, and to understand better the developmental changes in brain structure in both healthy and clinical populations. It is thus clear that neuroimaging methodologies available today provide the best way to understand *in vivo* human brain maturation.

Longitudinal studies are also more informative than cross-sectional measurements in showing the trajectories of structural brain development. In fact, many studies suggest that the developmental trajectory of the human brain may be considered an endophenotype [90, 91]. This is likely as many neurodevelopmental disorders originate from aberrations in early brain development [5, 6]. Thus, through the use of neuroimaging we can begin to characterize better maturational trajectories that hold promise for both identifying underlying pathophysiological mechanisms related to psychiatric illness, and for serving as potential biomarkers of risk that may be used for early identification and intervention.

As the field begins to move forward, technological advances will make possible still more refined interrogations into the underlying biological components reflected more indirectly by imaging measurements. Most importantly, the utilization of multi-modality imaging techniques will provide a more complete picture of both early and later cortical developmental patterns and also answer critical questions about the typical and atypical structural and functional networks that emerge throughout the lifespan.
